# Temporal Direction, Intuitionism and Physics

**DOI:** 10.3390/e26070594

**Published:** 2024-07-11

**Authors:** Yuval Dolev

**Affiliations:** Department of Philosophy, Bar Ilan University, Ramat Gan 5290002, Israel; dolevyu@biu.ac.il

**Keywords:** direction of time, time-reversal invariance, thermodynamics, statistical mechanics, metaphysics of time, presentism, eternalism, tense and passage, intuitionism, determinism, indeterminism, open future

## Abstract

In a recent paper, Nicolas Gisin suggests that by conducting physics with intuitionistic rather than classical mathematics, rich temporality—that is, passage and tense, and specifically the future’s openness—can be incorporated into physics. Physics based on classical mathematics is tenseless and deterministic, and that, so he holds, renders it incongruent with experience. According to Gisin, physics ought to represent the indeterminate nature of reality, and he proposes that intuitionistic mathematics is the key to succeeding in doing so. While I share his insistence on the reality of passage and tense and on the future being real and open, I argue that the amendment he offers does not work. I show that, its attunement to time notwithstanding, intuitionistic mathematics is as tenseless as classical mathematics and that physics is bound to remain tenseless regardless of the math it employs. There is much to learn about tensed time, but the task belongs to phenomenology and not to physics.

## 1. Introduction

The premise of this paper is simple, though controversial: the nature of time cannot be fully captured by either mathematics or physics. While physics provides theories about occurrences *in* time, it cannot address the essence of time itself. Temporal asymmetry, specifically the difference between futurity and pastness, which is so central to our experience of being in the world, is bound to remain outside the domain of physics and mathematics. On the positive side, phenomenology offers a wealth of insights into the nature of time—perhaps all the insights we may want.

Controversial, perhaps, yet these claims are not new. Recently, however, a novel suggestion was put forth for devising a formal framework wherein time and the future’s openness are integral. In a nutshell, the idea is to replace time-blind classical mathematics with temporally sensitive intuitionistic mathematics when doing physics. The claim is that physics conducted with this new mathematics will also be temporal in a way that physics as we know it is not.

However, I argue that, to the extent that temporality is part of intuitionistic mathematics, it is found there in precisely the same way that it has always been part of physics—as an accompaniment that is indispensable but *external* to physics, as part of the language and conceptual background that scientific research presupposes and within which it takes place. Thus, as far as having experienced temporality expressed in physics is concerned, the proposed replacement of the mathematical framework leaves matters as they were.

The upshot is that time and specifically the temporal asymmetry between the past and the future remain something for which, as Einstein put it, “physics has no possibility of expression” (in a 1952 letter to Ms. Levitova). Tensed temporality turns out to be a matter for phenomenological rather than scientific research. The implications for physicalism are clear and immediate. However, there are further implications for the current preoccupation with the potential sentience of machines.

## 2. The A-Temporal Nature of Classical Mathematics

Two facts about classical calculus must be considered when its application in physics is analyzed: it is a-temporal and it deals with infinities. Both these facts are important for understanding how time figures in physics and both are incongruent with the reality physics is concerned with, which is temporal and finite. 

In an important paper, “Indeterminism in Physics, Classical Chaos and Bohmian Mechanics: Are Real Numbers Really Real?”, Nicholas Gisin [1] shows how the prejudice that physics is deterministic follows from a reliance on real numbers and real (and complex) analysis in describing reality. Real numbers, with the exception of countably many of them, are infinite entities that cannot have a finite representation (the rational numbers are countable and, of the irrational ones, again, only countably many can be represented by finite symbols such as π or $\sqrt{2}$). In a nutshell, Gisin’s argument is that the contention that the equations of motion are deterministic, namely, that, given a system, its state at *t*_1_ is uniquely determined by its state at *t*_0_, presupposes that the system’s state at *t*_0_ can be described with infinite accuracy. However, that is never the case. Given the laws of motion, if a system’s state at *t*_0_ is described only with finite accuracy, then that state is compatible with a multitude of states at *t*_1_. In his paper, Gisin shows how the supposed determinism of classical mechanics is achieved by formulating it with infinite entities—the real numbers—in much the same manner that quantum physics is rendered deterministic in Bohmian mechanics by means of hidden variables.

This—the removal of an *open* future from its portrayal of reality—is the first divergence of physics from experienced temporality. However, matters are worse, for the fact that classical mathematics is a-temporal entails that physics does not capture any notion of futurity, open or not. The same is true of course for presentness and pastness. To quote Einstein in full this time, “physics … has no possibility of expression for ‘now’ (present), for ‘past’ and for ‘future’”. Given the state of a system at *t*_0_, the laws of motion enable establishing its state at any other time t1. However, these laws and, indeed, nothing in the theory, tells where the system is *now*. Being told the precise time of a solar eclipse (e.g., 29 March 2025) will not enable you to see it, unless you know what time it is *now*. And nothing in the theories of physics holds this information. Indeed, the irremediable absence of tense from physics is part of the reason why eternalism is so popular with philosophers of time that draw their understanding of reality from science.

However, physics’ tribulations with time are not over, for it is not clear that even the tenseless earlier/later dichotomy, which expresses a much thinner conception of temporal directionality than the one embodied by tense and passage (by passage I mean the becoming of future events present and of present ones past), is captured by physics. In the above references to the state of a system at *t*_1_ it was not stated, nor did it have to be stated, whether *t*_1_ was earlier or later than *t*_0_. The natural numbers are no more temporally arranged when they serve as indexes for *t* as they are when used to count apples (that the laws of nature are so-called “time reversal invariant” is one expression of this fact.).

Without the second law of thermodynamics, physics cannot make this distinction. However, even with this law, in its thermodynamic, not mechanical statistical form, what we obtain is a directed sequence of events, not necessarily one that is correlated with temporal direction. Moreover, in its statistical form, the question of the direction in which systems evolve becomes a matter of probability, and this is certainly at odds with temporal direction, which exhibits no such sensitivity to statistics.

To sum up, physics is mute when it comes to time: it cannot express tense and passage, knows nothing of the future and of its openness, has no means to represent which moment or which event is taking place now, and does not have within its vocabulary the asymmetry between before and after, the static, tenseless form of temporal directionality. In addition, and relatedly, physics seems to give credence to determinism. And the source of its limitations seems to be the a-temporal, infinite mathematical language its theories are construed with (My study [2] is devoted to a detailed discussion of the absence of tense and passage from physics. It also discusses the so-called tenseless relations and claims that, given how these depend on tense and passage, they too are not part of physics. My study [3] concerns the second law of thermodynamics. I show there that it assumes temporal directionality rather than ground it.).

This line of reasoning has led Gisin to the idea that a different mathematical language, one closely tied to time and which shuns the problematic, unconstructive infinities of classical calculus, could alter matters significantly and empower physics to incorporate temporality like it never did before.

## 3. Intuitionistic Mathematics

The framework that is supposed to facilitate this advancement is intuitionistic mathematics, which, as Gisin puts it, “allows one to re-enchant physics, introducing a model of an objective “creative” time, i.e., a dynamical time that allows for an open future and the passage of time” [4].

The hallmark of intuitionism is that, in contrast with realism/Platonism, which takes mathematical entities to be outside of time and immutable, the objects of intuitionistic mathematics evolve as time passes. Intuitionism seems to have the openness of the future built into it: at any given moment the domain of mathematical objects consists only of what has hitherto been constructed, with more to be added as time goes on. The openness of the future consists in the fact that, at present, *there is no fact of the matter* as to what will be added. Formally, the law of excluded middle (henceforth, LEM) does not apply to propositions describing these future elements.

Brouwer’s choice sequences are a quintessence of the constructivist, indeterminate understanding of mathematical objects. Here is Posy’s description of them:

A choice sequence, σ, is given by a preset finite initial segment, σ(1), …, σ(k), together with a rule that, given (1), …, (k), determines the range of possible values for σ(k + 1) and onward. The rule might allow but a single value of σ(k + 1) for each *k*. That is an algorithm. But it might very well allow a broad collection of possible values. Given σ(1), …, σ(k), the set of available values of σ(k + 1) will be fully calculable ([5], p. 27).

Indeterminacy enters the picture with the fact that before a “creating subject” makes the choice, it is not determined which of all possible values for the next element of the sequence will actually become the sequence’s continuation (To be accurate, indeterminacy having to do with a creating subject is an intermediate between the indeterminacy found in absolutely free-choice lawless sequences and lawlike, algorithmic sequences. For an elaboration see Posy (2020), a superb introduction to intuitionism, in which it is presented in all its depth and thrill. Brouwer’s “creating subject” is discussed in Posy ([5], p. 28).). It is worth noting the striking resemblance this bears to the standard understanding of quantum mechanics, in which the wave function represents all possible outcomes of a future measurement, but, prior to the measurement, there is no fact of the matter as to which result will actually be obtained. That is determined only when the measurement, the parallel of the choice made by a “creating subject”, is made.

Intuitionistic mathematics, then, appears to be genuinely temporal, with an open, indeterminate future figuring among its constitutive elements. It is exactly the kind of mathematics, which, if rendered adequate for physics, would emancipate physics from determinism and from its timelessness.

Alas, the hope for temporalizing physics in this manner is short lived. As an evolving system that develops over time, intuitionistic math is indeed temporal, but only in a limited manner. To see what this means, let us begin by noting that intuitionism can, without omission of any content, be formulated tenselessly; that is, it can be fully articulated with no use of the word “future”, and so with no reference to the future’s openness.

As just noted, choice sequences are the embodiments of constructions in time. At some initial moment *t*_0_ the sequence begins with a preset segment, and then, with time, it grows: at *t*_1_ an element s_1_ is added, at *t*_2_ another element *s*_2_, *s*_3_ at *t*_3_, and so on. At *t*_1_ it is undetermined which of all possible values for s_2_ will in fact be chosen, that is, at *t*_1_ the law of excluded middle does not apply to the proposition “*s*_2_ is Ω” (Ω being a member of the set of values *s*_2_ can assume), hence the indeterminism. However, notice that in this formulation there is no mention of the future. We can say that at *t*_1_ the later moment *t*_2_ is future. We can *say* that the sequence “grows” as time passes. However, these appeals to tense and passage are just a manner of speaking. They add nothing to what has already been stated in a tenseless language.

A comparison with the tenseless analysis of motion can help clarify how in this case the employment of tensed language only covers up the fact that the thesis being put forth is tenseless through and through. Eternalists do not deny the reality of motion, that, for example, there are busses that travel from Boston to NY. However, for eternalists, that only means that, focusing on some particular bus, at *t*_1_ the bus is in Boston, at a later time *t*_2_ it is in Harford, etc., until finally at *t_k_* it is in NY. In this analysis of motion (known as Cambridge, or, sometimes, Russell motion) there is no passage, no past, present, or future. It is important to note that eternalists do not shun tensed language—it is standard linguistic practice, when the bus is in Hartford, to say that it *will* get to NY in three hours. The so-called “New B-theory” was devised precisely for the purpose of reconciling eternalism with the fact that the tenses are not removable from language and are not dispensable. The point is, however, that that is where the tenses are found—in language and psychology, not in the world.

Intuitionism works with the exact same tenseless conception of change and evolution. That the tenses are used while laying it out does not mean they have a role in it. Intuitionists can avow their conviction that mathematical structures develop with time and make reference to time’s passage, but these uses of tense are optional add-ons that can be dispensed with and do not render intuitionism tensed any more than the employment of tense by eternalists (“the bus will reach its destination in one hour”) makes their theory of motion tensed. For intuitionism to be tensed, the tenses have to be indispensable to it, to be irremovable from its propositions, which they are not. To the contrary, the now is absent, and cannot be inserted into it. *We* know what the state of things is now, e.g., where the bus is now, or how many digits in the decimal expansion of π are currently known. However, theories can only tell us how things stand *at a given moment t*; they cannot tell us whether *t* is present, or past, or future. What time it is now, today’s date, is not information that we can glean from, or insert into, any theory.

Posy states that intuitionism’s temporality is deeply tensed and therefore nondeterministic: “Ips’s [infinitely proceeding sequence, such as choice sequences] show this. Grasping an ips α is the paradigm temporal experience, a clear sense of past, present and future. a’s fixed initial segment is the clear past. The information we have at $\Sigma_{0}^{a}$ (the moment of grasp) defines the present. And the rule for choosing α’s further elements is what we can say of the future. And that future is palpably indeterminate: the rule need not be an algorithm” ([5], p. 81).

However, far from establishing intuitionism’s temporality, what this passage offers is a glowing instance of how a superficial glaze of tensed vocabulary can create the illusion that a theory is tensed while in truth it is thoroughly tenseless. Just note how readily this passage can be rephrased so that no mention is made of the past, present, or future. All the passage says is that grasping an ips α at a given moment *t* consists of grasping an initial segment which is already fixed before *t*, as well as information added at *t*, and a rule for choosing α’s further elements at subsequent moments. No allusion to a “clear past”, a present, or a future is required. We could call the later moments *future* if we so chose, but that is just like calling the arrival of the bus in NY “future”—a use of words that would not for one minute shake an eternalist’s conviction regarding the tenselessness of motion.

In intuitionism, just as in Cambridge change, temporal evolution is captured by the relativization of a property to a *tenseless* moment in time. In the case of motion, the property in question is spatial location. For intuitionism, it is the possession of a truth value by a proposition. A truth value can be attached to a token at a time *t* in the way that a spatial location *x* is assigned to the bus at a time *t*. In both, tense and passage are completely out of the picture. Imagine that a proof of Goldbach’s conjecture is found in 2050. The conjecture’s status at that moment changes from being neither true nor false to being true. That is what happened to Fermat’s conjecture in 1995. However, the entire sequence of events regarding Goldbach’s conjecture can be given tenselessly: in 1742 Christian Goldbach puts forth the conjecture that every positive even integer can be written as the sum of two primes. In 1924 Hardy and Littlewood show that… in 1930 Lev Schnirelmann shows that…. In 1951 Yuri Linnik proves that…in 2050 so-and-so proves the conjecture. Again, before 2050 the conjecture lacks a truth value, while from 2050 onwards it possesses a truth value. Can a Martian who is given this information know whether the conjecture has been proven or refuted, whether it *now* has a truth value? Only if they know what date it is today, but this piece of information has to be provided in addition to the above chronology of the conjecture’s evolution.

The analogy between Cambridge change and intuitionism may be objected to. It could be claimed that spatial locations and truth values are categorically very different. The former are properties of material objects, the latter are rather abstract properties of propositions. The change a proposition undergoes is from lacking a truth value to possessing one, whereas a bus in motion always has a spatial location. However, this difference is irrelevant in the present context. The similarity is important and consists in a change in properties, which is given in tenseless terms, without the *now* figuring in its depiction. This both cases share.

## 4. How Time Figures in Intuitionism and in Physics

I should emphasize that I take the tenseless theory of change and motion to be incoherent (my reasons are presented in several publications.). Regarding mathematics, my leaning is towards realism. My aim here, however, is not to present objections to intuitionism, only to clarify in which manner exactly it incorporates temporality. I want to show that the time that figures in it is tenseless time, just as in the tenseless theory of motion. One consequence of this will be that even if physics is reformulated with intuitionistic mathematics (what this could mean will be discussed shortly), tense and passage are still external to it.

The tenseless character of intuitionism is harder to detect, for, unlike eternalism, which celebrates tenselessness and proudly highlights the contrast between the tensed nature of the experience of motion and what the theory contends is the truth—namely, that motion is a tenseless affair—intuitionism purports to be a theory in which rich, tensed temporality figures centrally in both the epistemology and the ontology of mathematics.

However, the deeper reason why the tenselessness of intuitionism is evasive is that *we* are in tensed time, living in the present, with a past behind us and a future before us. Regardless of our metaphysical convictions, we cannot but experience, think, and speak of events and occurrences as located in either the past, present, or future. As committed as we may be to eternalism and/or determinism, the sun’s rising tomorrow morning cannot but be apprehended by us as *future*. If we think of tomorrow’s game, we cannot but experience its futurity as *open*. An eternalist, who is writing a paper establishing that tense is an illusion, cannot but experience that activity as occurring in the present and the paper’s publication as future. Tense imbues our cognition, experience, and language through and through. Our natural, pre-reflective stance is that things—our experiences and thoughts as well as the events we experience and think about—are in tensed time. Tenselessness is foreign to our thinking and experience, it is so far removed from them that it is far from obvious that we can wrap our heads around it—in any context. It takes effort and argumentation to so much as suggest that motion is not in tensed time. Moreover, when a theory comes along purporting to couch mathematics in time, the time figuring in it will be natural time, time as we know it from experience—flowing and consisting of a past, present, and future. We know no other time. Other than as a construction of philosophers, we do not know what tenseless time is. We may resist the temporalization of math, but to the extent that we entertain the possibility, the time figuring in this attempt will be time as we know it—tensed and passing. It would be all but expected that when Posy lays out the principles of intuitionism, he will speak of “a clear past, present and future”.

But there is no tense or passage in the time of intuitionism. We project our notion of time on the temporality of intuitionism. However, like in a failed organ transplant, intuitionism cannot absorb time. Time as we know it remains external to it. *We* cognize the development of a choice sequence as something with a past, present, and future, but this temporality resides in the world and in how we experience it. It is no more part of intuitionistic mathematics than it is of Newtonian physics. The tenseless theory of time came into being in the beginning of the 20th century in the work of McTaggart [6] and then received a significant boost with the appearance of Einstein’s relativity theory. Before that, no one thought of physics as tenseless, yet physics was always tenseless—tense and passage were never part of it. It is just that, in the absence of a tenseless theory of time, no one thought of physics as tenseless. The distinction between experienced time and time as it figures in a theory was not made explicit or noticed. Similarly, no such distinction was made regarding intuitionism. However, rich temporality is external to intuitionism just as it is to Newtonian physics.

Contrast this with tenseless relations. A line—the time coordinate—can represent these relations. The distance between the tick marks on the line represents a time interval of a certain duration (That tenseless relations can be represented spatially goes hand in hand with other elements in the spatialization of space that are integral to eternalism.). But what could a representation of tense and passage look like? How could it be achieved? No diagram of phase space comes with a moving dot that indicates the state of the system *now*. There is no, and cannot be, an equivalent of such a dot in any theory of physics (A fact that “worried him seriously”, as Einstein confessed to Carnap [7], p. 37–38.). So, what could turn the trick? Merely being situated in time, perhaps even with an open future, is not enough. There is a difference between being something and representing that thing. An emoji can represent happiness but is not itself happy, while Jane is happy but is not a representation of happiness. Say we follow Anscombe and think of a Galton board as a system with an open future—Anscombe persuasively makes the point that viewing the board as a system whose trajectory is deterministically necessitated in advance is a matter of choice, not a position imposed on us by physics or by logic or by conceptual analysis. Still, even if the board evolves into an open future, it instantiates time’s passage, it does not represent it. Intuitionism is a philosophical theory that says that the body of mathematical facts and knowledge grows with time’s passage and has an open future. In this respect, it is just like presentism that says of, say, a Galton board that it evolves with time’s passage and has an open future. However, the framework intuitionism is about—intuitionistic mathematics—does not represent tense and time’s passage any more than a Galton board does.

The source of confusion is found in the conflation of intuitionism as *a philosophical theory* about math and intuitionistic mathematics. As a system of mathematics, it can be formulated in purely tenseless terms. Proofs of theorems in intuitionistic mathematics are written down in notebooks just like proofs in classical math. Nothing on the page in which such a proof is laid out represents time’s passage or the *now*. It is the philosophical doctrine that says *of* intuitionistic mathematics that it is in time, that it evolves as time passes, and that it has an open future, and the philosophical doctrine is external to the mathematical activity it speaks of, looking at it and commenting on it from the outside, as it were.

Gisin’s proposal is, of course, not to replace classical math as it figures in physics with a philosophical doctrine of intuitionism but with intuitionistic mathematics itself. However, that would amount to replacing one tenseless mathematical formalism with another that is just as tenseless. True, of this formalism it can be said that it is the outcome of an activity that takes place in tense time, but that is of no help insofar as inserting tense into the theories of physics is concerned, because the mathematical formalism figuring in the theories continues to be tenseless.

To sum up, that there is a philosophical doctrine that views the mathematical landscape as evolving with time’s passage does not facilitate inserting time’s passage into physics. Tense and passage, central to the reality physics describes, remain external to it, no matter with which mathematical framework the theory is formulated. That, of course, does not mean that tense and passage are not part of reality. Physics is utterly compatible with tense and passage, but they are not part of it.

Theories in general, and those of physics in particular, can impart much temporal information, but they cannot tell us what of all that is described in them is happening now. The *now* is an experiential given, which cannot be read off from any device, let alone from any theory. Even a clock does not tell that it is now, rather, it tells that it is now 5 pm, that is, it conjoins a calendrical time with a now that is already apprehended experientially.

That tense and passage belong to the fundamental structure of reality was not even questionable prior to the appearance of eternalism. There were no eternalists in the 18th and 19th century, and the thought that tense and passage were not part of reality did not occur to any physicist back then. It is just that tense and passage resided in how people spoke, experienced, thought, and behaved, not in their theories. The development of intuitionistic mathematics did not change this, it did not bring with it a novel manner of relating physics to tense and passage. Also now, the only way to “temporalize” physics is by an external indicator, a laser pointer, for example, which superimposes the present on the appropriate location in a coordinate system.

In any theory, time is represented by a tenseless timeline. It is incumbent upon us to infuse it, from the outside, with temporality—we can always highlight a point on a timeline and say that is where the present is located, but this extra act remains external to the representation. The same can be done with intuitionism. A diagram in which the various possibilities for the next element in a sequence are shown to branch off from the last element added to the sequence supposedly depicts the future’s openness, but here again tense and passage are utterly absent.

## 5. The Future’s Openness

That the future is open is not important in itself. The significance of the future’s openness is tied, for us, to deliberation, often, to the need to choose between possibilities. However, this notion of possibilities is a longstanding conceptual challenge.

Before discussing it briefly, the meagerness of intuitionism’s temporality should be highlighted from another angle. Even if in intuitionistic mathematics the future is open, that does not immediately entail that intuitionistic mathematics knows of multiple future possibilities. That Goldbach’s conjecture lacks a truth value on 1 June 2022 does not logically necessitate that there is a possibility that it will be refuted at some moment later than 1 June 2022. In general, that at *t*_1_ proposition *p*, which speaks of an event that takes place at a later moment *t*_2_, lacks a truth value does not presuppose or dictate that there are multiple possibilities for what may happen at *t*_2_. A proposition may lack a truth value in the way a tree lacks leaves in the winter: at a later date the tree will grow leaves and the proposition (or, to be accurate, a later token of it) will attain a truth value. That things “change” in this manner does not mean they could have changed in a different way. On 1 June 2022 there is neither a proof of Goldbach’s conjecture nor a counterexample, and so it lacks a truth value, but, assuming a proof is produced at a later date, that on 1 June 2022 there is a genuine possibility of finding a counterexample is a further claim, which is not entailed by the mere absence of a truth-value. Intuitionism holds that mathematics evolves with time, but that is not tantamount to ascertaining that there really are multiple alternative futures. If so, the gap between intuitionism and eternalism is smaller than it may appear to be.

This last observation brings us back to the limitations on representing time, limitations stemming from issues concerning the ontology of possibilities. Future possibilities, do they exist? In what manner? Do they co-exist in the future, side by side, as it were? Sometimes that is how they are depicted, as lines branching out from a point—“the present” (Bergson’s [8] scathing criticism of this picture is as valid today as it was when he made it.). Picturing these future possibilities in this manner begs the question—how is the one that will end up being actualized different from those that will not? Those that will not become actual will never come into being, and they are not part of reality now. So, in what sense do they exist at all?

These ancient questions cannot be broached here. The point is that, no matter how we think they should be approached, it is plain that answers will not come from representations of physical systems or from mathematical frameworks. Nor would the stance we end up adopting about the nature and essence of possibilities be something we could incorporate into these representations. The future’s openness is outside of physics and is bound to remain external to it. That leaves us with two options. If, in principle, physics encompasses every aspect and facet of reality, then there is no such thing as an open future. Otherwise, physics does not cover every aspect and facet of reality. I think that, to many, this is self-evident. So much of what we care about—love, humor, music, normativity—is not part of the language of physics, perhaps not part of science at all. This is certainly a source of concern and unease if you’re a reductive physicalist, but only then.

Let us introduce a distinction between what can be dubbed *flat* and *consequential* indeterminism. Flat indeterminism is tenseless and does not assume an open future. It consists simply in the existence, at any given time *t*, of propositions to which LEM does not apply. Consequential indeterminism is tensed and is tied to the future’s openness. Insofar as freedom, responsibility, creativity are concerned, only consequential freedom matters.

Thinkers such as Gisin are not after tenseless indeterminacy. Tenseless indeterminacy is just as much a disappointment as tenseless motion, which is useful for mathematical representations of motion but is a far cry from experienced motion. Tenseless indeterminacy lacks what makes real indeterminacy important—an open future. Tenseless motion and tenseless indeterminacy are alike frozen, fixed, and hollow.

We care about indeterminism because of future events that are important to us—the game’s final score, the results of the next election, the future of our planet. Our *hope* that things will evolve favorably is rooted in knowing that that is a genuine possibility, albeit one among many. Tenseless indeterminism, in contrast, makes us yawn.

Gisin’s arguments regarding determinism are not merely about the kind of mathematics suitable for physics. In fact, they are not about that at all. It is not as though he is advocating to replace classical with intuitionistic mathematics in the actual notebooks in which physicists perform their calculations and develop their ideas. His true interest is in reinstituting, let us call it, Humanism, in the Jamesean sense, (or Bergsonean, or Husserlian or Wittgensteinean sense), into our conception of the world, and to do so against a powerful current that has been dominating large portions of the philosophical landscape for more than a century, a central principle of which is that our conception of the world should be shaped first and foremost, not to say exclusively, by science. Determinism has been regarded as a fundamental element of this conception. Gisin’s motivation is to show that physics has been wrongly portrayed, often by some of its leading practitioners, as lending unshakeable credence to determinism. Moreover, his pushback against determinism is fueled by his conviction that we humans are free. Physics, he shows, does not impose on us a view according to which the world is inhospitable to free will and free action. It does not drive a wedge between how we experience ourselves as actors in the world, and what we are supposed to believe about it. In other words, Gisin’s argument is not about technicalities and not about theory. It is about the nature of the human being and her place in the world. It is about rehabilitating respectability, after a century of positivistic scientism, to a conception of reality that sees the world as a place in which we are striving to lead a good life and, hopefully, a life of goodness. The openness of the future is central to this care, and that is the meaning of indeterminism.

However, Gisin wants more than just to show that physics is not inherently deterministic. He would like physics to do much more, to explain “how time passes” ([4], p. 13366) and to register the present’s “thickness” ([4], p. 13365), as if passage is a physical phenomenon like a rainbow and presentness some kind of stuff the thickness of which can be measured. Elsewhere in his paper, Gisin expresses his grievances with Dolev’s contention that “tense and passage are not, never were, and probably cannot be part of physics and its language”. Gisin asks, “Shouldn’t one reply to Dolev by adapting the mathematical language used by physics to make it compatible with indeterminism?” ([4], 13346-7). As discussed above, physics has always been compatible with experienced temporality. Compatibility, however, is significantly more modest than Gisin’s more ambitious aim of a physics of tense and passage—in his words, a physics that can “tell stories of *how nature does it* in a language that allows humans to gain intuitive understanding” ([4], p. 13346-7, italics in original). That is a fantasy—physics cannot tell stories about what is irremediably foreign to its vocabulary.

In a thought-provoking 2024 paper [9] co-authored with Flavio Del Santo devoted to an analysis of the manners in which time figures in logic, mathematics, and physics, Gisin reiterates his conviction that physics can and should “allow one to tell stories about ‘how nature does it’”. One could, perhaps, claim that whether tense and passage are part of the story of physics depends on what one takes the scope of physics to be. If it includes physicists’ sense that time passes, their verbal confirmation that time passes, if it includes what physicists think the world their theories are about is like, then yes, tense and passage are part of the story of physics. However, then, of course, they were already part of the story told by Newtonian physics. If, on the other hand, physics is what it is standardly taken to be—what we find in books, scientific papers, in the mathematical equations of the laws of nature, in the results of experiments, or in the theories of physics themselves—there is no trace of passage there.

Gisin claims that the approach advocated by Del Santo and himself “leads to a naturalistic characterization of the difference between past, present and future”. However, it is not clear from their papers what this is supposed to mean. If it means that in physics one can find a characterization of passage and tense in the same way that the laws of nature can be found there, the above discussion shows that is not the case. If the idea is that physics will, in some other way, tell us something about, enrich our understanding of, passage or the distinction between the past, present, and future, no such enrichment is forthcoming. To see how a fruitful investigation of tense and passage can be performed and what results it can yield, one needs to look at the works of phenomenologists such as Husserl and Merleau-Ponty. Studying attempts such as theirs to understand the essence of presentness, futurity, and pastness, one is immediately struck by how far removed these investigations and their insightful outcomes are from anything that can be gleaned from physics.

However, it is not just phenomenology. In the world of analytic philosophy, Dummett’s work on intuitionistic logic and his rejection of bivalence as a fundamental principle of logic have been immensely influential. No one has advocated the replacement of classical logic with intuitionistic logic and the rejection of the law of excluded middle and bivalence more vehemently than him. However, nowhere did he claim that merely removing bivalence from logic makes for a formalism that “tells the story” of tense and passage. Principles such as modus ponens and A∧B→B, are part of intuitionistic logic, passage is not. It is *we*, who live in time and experience its nature, that assert that for our logic to cohere with reality our understanding of logic must allow that there are propositions to which bivalence does not apply. *We* can say that there is an open future, we can say, as Aristotle did, that bivalence does not apply to future contingents. However, at any given moment, which propositions are to be put in the future tense, which propositions bivalence applies to and which not, above all, *what it means to be future*, or for the future to be open—these are matters about which logic, classical or intuitionistic, has absolutely nothing to say. To repeat, for that, we need to turn to phenomenological investigations, or to Bergson’s, let us call it, experiential philosophy. However, again, Bergson, from whom Gisin borrows the term “creative time”, never tired of stressing that the temporality he was studying, *lived temporality*, contrasted sharply with time as it is found in physics. One recurring theme of these phenomenological/experiential excursions is that temporality cannot be divorced from normativity. Therefore, unless normativity becomes part of the story physics tells, rich temporality, tense, and passage are not part of the story either.

In sum, unless you are a reductive physicalist, such a fantasy should not appeal to you. Giving up on analyzing tense and passage scientifically does not mean relinquishing the aspiration to attain acute insights into their nature. Time can be richly and profoundly explored through a study of its role in our lives, a project phenomenologists have been engaged in for decades.

## 6. Conclusions

It takes someone in the unique position of Gisin, a theoretician and a world-leading experimentalist, to have the kind of insight into the application of mathematics in physics that leads to the understanding that determinism is not integral to the theories of physics but rather belongs to certain interpretations of these theories, interpretations which are shaped by the fact that physics is conducted with what Gisin calls infinite information numbers, namely, the real numbers of standard calculus. These numbers can, supposedly, represent location with infinite accuracy, and therein lies the supposed potential for predictions that are infinitely accurate. Gisin’s penetrating insight is that this is a myth—in practice, no theory, no computer, no brain processes infinite information. This means that no matter how accurately we describe the state of a system, that state is compatible with an array of states the system may be in at other times. However, the further step Gisin takes, of claiming that a different mathematical framework will do the impossible and give mathematical expression to tense and passage, will facilitate absorbing indeterminism into physics, is futureless.

In passing I note (though this requires a more extensive discussion) that the same can be said of machines that are the product of, among other things, physics. They cannot be regarded as representing, let alone experiencing, tense and passage. If this remark is correct, it raises a serious question regarding the validity of attributing sentience to machines. For us, tense and passage, and consequential indeterminism, are essential elements of experience. If these facets of experience cannot be recognized in machines, which, so it seems, must be the case given that they cannot be recognized in the physics and mathematics machines are based on, then machines cannot be thought of as experiencing in any way that resembles human experience.

The quest to conceptualize indeterminism, not as an attitude to the world and life which is compatible with physics, but as integral to physics itself, as the flesh and bones of its mathematics, is, I believe, the product of a humanistic temperament trapped in a scientistic environment. However, rather than pursuing it, it is possible to simply acknowledge the irreducibility to theory of the experience from which our understanding of time derives. That, to borrow from James ([10], p. 146), can be performed as an exercise of one’s untheorizable freedom (“…our first act of freedom, if we are free, ought in all inward propriety to be to affirm that we are free” ([10], p. 146).).

## Data Availability

No new data were created or analyzed in this study. Data sharing is not applicable to this article.
